# Single-nucleotide variants in human CD81 influence hepatitis C virus infection of hepatoma cells

**DOI:** 10.1007/s00430-020-00675-1

**Published:** 2020-04-22

**Authors:** María Pía Alberione, Rebecca Moeller, Jared Kirui, Corinne Ginkel, Mandy Doepke, Luisa J. Ströh, Jan-Philipp Machtens, Thomas Pietschmann, Gisa Gerold

**Affiliations:** 1 Institute for Experimental Virology, TWINCORE, Centre for Experimental and Clinical Infection Research, a joint venture between the Medical School Hannover and the Helmholtz Centre for Infection Research, Feodor-Lynen-Straße 7, 30625 Hannover, Germany; 2 Institute of Virology, Hannover Medical School, Hannover, Germany; 3 Institute of Biological Information Processing (IBI-1), Molekular- und Zellphysiologie, and JARA-HPC, Forschungszentrum Jülich, Jülich, Germany; 4 Institute of Clinical Pharmacology, RWTH Aachen University, Aachen, Germany; 5 Department of Clinical Microbiology, Virology and Wallenberg Centre for Molecular Medicine (WCMM), Umeå University, Umeå, Sweden

**Keywords:** Hepatitis C virus, HCV, Hepatocyte, Tetraspanin, CD81, Receptor, Single-nucleotide variant, Entry, Genetic variant

## Abstract

An estimated number of 71 million people are living with chronic hepatitis C virus (HCV) infection worldwide and 400,000 annual deaths are related to the infection. HCV entry into the hepatocytes is complex and involves several host factors. The tetraspanin human CD81 (hCD81) is one of the four essential entry factors and is composed of one large extracellular loop, one small extracellular loop, four transmembrane domains, one intracellular loop and two intracellular tails. The large extracellular loop interacts with the E2 glycoprotein of HCV. Regions outside the large extracellular loop (backbone) of hCD81 have a critical role in post-binding entry steps and determine susceptibility of hepatocytes to HCV. Here, we investigated the effect of five non-synonymous single-nucleotide variants in the backbone of hCD81 on HCV susceptibility. We generated cell lines that stably express the hCD81 variants and infected the cells using HCV pseudoparticles and cell culture-derived HCV. Our results show that all the tested hCD81 variants support HCV pseudoparticle entry with similar efficiency as wild-type hCD81. In contrast, variants A54V, V211M and M220I are less supportive to cell culture-derived HCV infection. This altered susceptibility is HCV genotype dependent and specifically affected the cell entry step. Our findings identify three hCD81 genetic variants that are impaired in their function as HCV host factors for specific viral genotypes. This study provides additional evidence that genetic host variation contributes to inter-individual differences in HCV infection and outcome.

**Electronic supplementary material:**

The online version of this article (10.1007/s00430-020-00675-1) contains supplementary material, which is available to authorized users.

## Introduction

Hepatitis C is an infectious disease of the liver caused by hepatitis C virus (HCV) and is a major public health problem. The World Health Organization (WHO) estimates that 71 million individuals worldwide are chronically infected. Approximately 400,000 patients die annually because of complications of chronic HCV infections [1]. The clinical course of the infection is diverse: while 25% of the acutely infected patients achieve spontaneous clearance of the virus, 75% remain chronically infected. Chronically infected individuals if untreated may develop a progressive liver disease that can lead to hepatic decompensation due to liver cirrhosis and hepatocellular carcinoma. Unless the liver is replaced by transplantation, this may lead to death. Some patients, however, although chronically infected, remain asymptomatic for life [2]. These notable inter-individual differences in susceptibility and disease progression remain poorly understood. The study of genetic variations in the population could lead to important insights into the underlying mechanisms of the clinical observations.

HCV is a member of the genus *Hepacivirus* belonging to the family *Flaviviridae*. It is a small, enveloped virus with a positive-stranded RNA genome. HCV is hepatotropic and is classified into seven genetically distinct genotypes (GTs) [3]. HCV enters into hepatocytes in a coordinated multistep process, which involves sequential interactions between viral particles and several host cell-surface factors. These include the scavenger receptor class B type I (SR-BI), the tetraspanin hCD81, the tight junction components claudin-1 (CLDN1) and occludin (OCLN) [4–7]. Eventually, the viral uptake into the endosomes occurs through clathrin-mediated endocytosis [8]. The fusion between the viral envelope and the endosomal membrane leads to viral-RNA release into the cytosol, thereby completing cell entry of the virus.

Several genetic variations in host factors and in genes involved in the immune response affect HCV infection and the host’s ability to clear the virus. Some could explain the inter-individual variability in disease progression or in treatment response. For instance, the single-nucleotide polymorphism (SNP) rs12979860, upstream of IL28B gene which encodes the type III interferon, is associated with HCV treatment response and clearance [9–11]. With regard to HCV entry factors, *OCLN* has several coding non-synonymous SNPs, which differ between populations with minor allele frequencies ranging between 1 and 2.5% [12]. Three of the SNPs tested using HCV pseudoparticle (HCVpp) and HCV cell culture-derived particle (HCVcc) had no effect on OCLN functioning as HCV-entry factor. Furthermore, the SNPs do not modify direct cell-to-cell spread of HCV, which requires OCLN [12]. Two coding non-synonymous SNPs in the *SCARB1* gene that encodes SR-BI are associated with reduced HCV cell entry. Additionally, a non-coding variant (G allele in rs3782287) is linked to a decreased HCV viral load in patients [13]. Taken together, these findings suggest that coding and non-coding variants of *SCARB1* influence the HCV replication cycle.

Besides OCLN and SR-B1, hCD81 is an essential entry factor for HCV. hCD81 is a membrane protein which belongs to the tetraspanin superfamily. hCD81 is composed of four transmembrane domains, one short cytoplasmic loop, two short cytoplasmic termini and two extracellular domains: small extracellular loop (SEL or EC1) and large extracellular loop (LEL or EC2). hCD81 is involved in several physiological processes in different cell types including cell morphology, motility, signal transduction, adhesion, activation, proliferation and differentiation of immune cells [14]. CD81 also participates in many bacterial, parasitic and viral infections, such as *Listeria monocytogenes* [15], *Plasmodium* [16], influenza virus [17], human immunodeficiency virus [18, 19], human papillomavirus type 16 (Finke et al., this reference is a part of the same MMIM Special Issue) and HCV. In the case of HCV, the viral glycoprotein E2 interacts directly with the LEL of the CD81 protein in the early stages of HCV entry [5]. More than 60 coding non-synonymous low-frequency variants of hCD81 exist [20]. Seven *hCD81* variants are known to support HCV entry in vitro: six of them located in the LEL and one in the transmembrane domain 4 (TM4). The variants do not have an effect on direct cell-to-cell spread of HCV and on neutralization by different CD81 antibodies [21]. Apart from binding to HCV, CD81 is involved in protein–protein and lipid interactions, which are important for post-binding events in the HCV replication cycle.

For HCV entry, hCD81 interacts with the entry factors SR-BI and CLDN1 [22, 23], and forms a complex with the co-factors GTPase HRas, serum response factor-binding protein 1 (SRFBP1), CBLB and calpain 5 [23–25]. The regions outside the LEL (here referred to as a backbone) have a critical role in post-binding HCV entry steps [26]. Moreover, the CD81 backbone is involved in cholesterol coordination. The crystal structure of hCD81 revealed a cholesterol-binding pocket between the two C-terminal transmembrane helices. According to molecular modelling, the presence or absence of cholesterol in this binding site seems to modify the conformation adopted by the LEL domain, thereby modulating the activity of CD81 [27]. Importantly, a point mutation of the E219 residue of TM4, important for cholesterol binding, reduces infectivity of HCV [26]. This implies that direct cholesterol coordination by hCD81 is required for HCV infection.

Due to the important role of hCD81, the aim of this study was to investigate the effect of five non-synonymous single-nucleotide variants (SNVs) leading to amino acid exchanges in the backbone of hCD81 on HCV susceptibility. We expressed the different *hCD81* variants in Lunet N#3 cells, which have undetectable endogenous hCD81 levels [28], and characterized them by flow cytometry, Western blot and confocal microscopy to determine the expression. Finally, we performed HCV pseudoparticle (HCVpp) and HCV cell culture-derived (HCVcc) infection assays to investigate whether the hCD81 variants can function as HCV entry factors. Different inter-genotypic HCV chimeras bearing the glycoproteins from five of the major clinical HCV genotypes were included to investigate if hCD81 variants differentially influence infection. The results indicate that non-synonymous SNVs A54V, V211M and M220I in the backbone of hCD81 render hepatoma cells less susceptible to HCV infection in comparison to the wild-type (WT) hCD81. These findings further increase our knowledge on inter-individual differences in HCV host factors and the possible impact on the course of disease.

## Materials and methods

### Generation of hCD81 non-synonymous genetic variants

Gene fragments (gBlocks) encoding hCD81 carrying the respective mutated codons for the variants A213T, V211M, M220I and 5 SNVs, and a C-terminal tandem hemagglutinin (HA) tag were commercially synthesized by IDT (Integrated DNA Technologies, Inc., Iowa, USA). Vector-insert overlapping regions were added to both ends of the gBlocks by PCR using the primers hCD81_Gibson_for (5′-CGA TCA CGA GAC TAG CCT CGA GGT TTA AAC GCC ACC ATG GGA GTG GAG GGC TGC-3′) and HA_Gibson_rev (5′-GGG GGG CGG AAT TCC TGC AGC CCG TAG TTT CTA GGC GTA GTC GGG CAC-3′). The amplified PCR products were inserted into the pWPI_BLR lentiviral vector using Gibson assembly according to the manufacturer’s instructions (New England Biolabs, Ipswich, MA, USA).

The variants R36L and A54V were cloned by fusion PCR using primers carrying the respective single-nucleotide polymorphism: R36L-for (5′-TGT GGC TCC TCC ATG ACC CGC AGA-3′), R36L-rev (5′-TCT GCG GGT CAT GGA GGA GCC ACA-3′), A54V-for (5′-AGA CAA GCC CGT GCC CAA CAC CTT CTA T-3′), A54V-rev (5′-ATA GAA GGT GTT GGG CAC GGG CTT GTC T-3′), and the flanking primers HAHA-Gibson-r (5′-TCC TGC AGC CCG TAG TTT AC-3′) and hCD81-Gibson-f (5′-TTA AAC CTG CAG GCG CGC CG-3′). The PCR products were cloned into the pWPI_BLR lentiviral vector in the multiple cloning site between BamHI and SpeI restriction sites.

All the constructs contained a tandem HA tag fused to the C terminus by a Gly4SerGly linker. Inserts were confirmed by direct sequencing. Detailed cloning strategies are available upon request.

### Cells and reagents

The Lunet N#3 human hepatoma cell subclone and HEK 293 T cells were maintained in Dulbecco’s modified Eagle’s medium (DMEM) supplemented with 10% fetal bovine serum, 1% l-glutamine, 1% non-essential amino acids, 1% penicillin/streptomycin at 37 °C and 5% CO_2_. Cells that expressed the hCD81 variants were positively selected using 5 μg/ml blasticidin.

### Lentiviral transduction and generation of cell lines stably expressing hCD81 variants

To produce pseudoparticles, HEK 293 T cells were co-transfected with three different plasmids: pVSVg encoding for the G protein of Vesicular Stomatitis Virus, pCMV_ΔR8-74 as a packaging construct and one plasmid encoding for a specific hCD81 variant. To improve RNA transcription and consequently vector production, sodium butyrate was added 24 h post-transfection [29]. Supernatants containing lentiviral particles were harvested at 48 and 72 h post-transfection and filtered through a 0.45-μm pore-size filter. To stabilize the lentiviral particles and improve the efficiency of lentiviral gene transfer, polybrene [30] and HEPES were added to a final concentration of 5 μg/μl and 20 mM, respectively. Afterwards, Lunet N#3 cells, which have undetectable levels of endogenous CD81 [28], were transduced with lentiviral particles, incubated for 4 h at 37 °C and then 2 ml of fresh DMEM-complete was added. Selection for positively transduced cells with blasticidin commenced 48 h post-transduction.

### CD81 surface staining and flow cytometry

To measure the surface expression of hCD81, single-cell suspensions of the Lunet N#3 subclones were stained on ice for 30 min using APC-conjugated mouse monoclonal anti-hCD81 antibody (BD, JS-81, 10 μl per 1 × 10e6 cells in 50 µl final volume) or an isotype control antibody (BD, 10 μl per 1 × 10e6 cells in 50 µl final volume) diluted in PBS supplemented with 1% FCS. Afterwards, cells were washed with FACS buffer (PBS 1% FCS) to remove unbound antibodies and re-suspended in 150 μl fixation buffer (PBS 1% FCS 0.5% PFA). Fluorescence signals were measured by flow cytometry using the AccuriTM C6 Cytometer (BD) and FlowJo V10 Software was used to analyze data.

### Immunoblotting

Cells were washed with PBS, centrifuged, and the pellet was stored at − 20 °C. Afterwards, cells were re-suspended in 300 μl of lysis buffer (1% Nonidet P40, 10% glycerol, 1 mM CaCl_2_ in HEPES/NaCl) supplemented with protease inhibitor mix (Sigma #P8340, dilution 1:100). After 30 min of lysis on ice, nuclear debris was pelleted at 12000×*g* for 10 min at 4 °C, and the supernatant was transferred to a fresh tube and stored at − 20 °C. The total protein content in each sample was determined by Bradford assay. To perform the electrophoresis, the volume of sample containing 25 μg of proteins was mixed with 10 μl of 2 × non-reducing sample buffer [50 mM of 1.5 M Tris–HCl (pH 6.8), 0.02% bromophenol blue, 4% SDS and 12% glycerol] and milliQ water to a final volume of 20 μl. Samples were incubated for 30 min at 30 °C and transferred on ice. In total, 20 μl of each sample was loaded onto a 12.5% polyacrylamide-SDS mini-gel and the electrophoresis was carried out at 100 V for 1.5 h. Proteins were transferred to a polyvinylidene difluoride membrane (PVDF) using a semi-dry blotter. The membrane was blocked for 1 h in PBS supplemented with 0.5% Tween 20 (PBS-T) and 5% milk. The membrane was then incubated with primary antibodies diluted in PBS-T supplemented with 1% milk (1.25 μg/ml for mouse anti-human CD81 clone JS-81 and 0.2 μg/ml for rabbit anti-GAPDH). After overnight incubation at 4 °C, the unbound antibody was removed by washing with PBS-T. The membrane was then incubated for 1 h with horseradish peroxidase-conjugated secondary antibodies (anti-mouse and anti-rabbit immunoglobulin G) diluted at 1:20,000 in PBS-T supplemented with 1% milk. After extensive washing, the bound antibodies were detected with the ECL Prime Western blotting detection system (GE Healthcare UK, Buckinghamshire, UK) according to the manufacturer’s instruction. The proteins were visualized using the ChemoStar Professional Imager System (Intas).

### Immunofluorescence analysis and confocal microscopy

To study hCD81 variant expression patterns, immunofluorescence staining followed by confocal microscopy was performed. To that end, hCD81 variant-expressing Lunet N#3 cells were seeded on poly-l-Lysin-coated cover slips in 24-well plate at a density of 5 × 10^4^ cell/well and incubated at 37 °C. After 24 h of incubation, cells were fixed for 20 min with 3% paraformaldehyde (PFA) at room temperature followed by washing with PBS. The cells were permeabilized using 0.1% Triton X-100 in PBS for 5 min. Afterwards, the cover slips were blocked with PBS/0.5% BSA for 10 min and incubated at 4 °C overnight in the presence of primary antibody (Mouse anti-human CD81, BD clone JS-81, 10 μg/ml, diluted 1:50 in PBS/BSA). Unbound primary antibody was washed off with PBS and the cells incubated with secondary antibody (goat anti-mouse-IgG-Alexa 488 diluted 1:1000 in PBS/BSA) for 1 h at room temperature in the dark. Following a washing step, nuclei were stained with DAPI (diluted 1:10,000 in H_2_O) for 1 min in the dark. Residual dye was removed by washing the coverslips with H_2_O. The cover slips were then mounted on glass slides using 7 μl of ProLong ^®^ Gold Antifade (Thermo Fischer). Immunofluorescence analyses were carried out using inverted Olympus IX-81 confocal microscope and the FV1000 Viewer (Olympus).

### HCV pseudoparticle infection

HCV pseudoparticles (HCVpp) were generated as was described before [24] by co-transfecting three different plasmids into HEK 293 T cells using polyethylenimine (PEI). The plasmids were a packaging construct (pCMV_ΔR8-74), a Firefly luciferase reporter plasmid (pWPI_F-Luc) and an expression vector encoding viral envelope proteins, either the HCV glycoproteins E1 and E2 of strain H77 or the G protein of Vesicular Stomatitis Virus (VSV-G). As a negative control, a pcDNA plasmid encoding no viral glycoprotein was also included to generate pseudoparticles. Pseudoparticles were harvested 48 and 72 h post-transfection by filtering supernatants through a 0.45-μm pore-size filter. As described above, HEPES and polybrene were added to stabilize the pseudoparticles, which were directly used to infect target cells.

Lunet N#3 cell lines expressing hCD81 variants were seeded into 24-well plates at a density of 3 × 10^4^ cells per well in media omitting blasticidin. The cells were transduced 24 h later using 1 ml of the pseudoparticle preparation. The VSV-G pseudoparticles were pre-diluted (1:100) to ensure comparable luciferase signals in all analyzed experimental conditions. After 4 h of incubation, pseudoparticles were removed, DMEM-complete was added, and cells were subsequently incubated at 37 °C. 72 h post-transfection, cells were washed with PBS and lysed with 200 μl luciferase lysis buffer (1% Triton X-100, 25 mM glycinglycine (pH 7.8), 15 mM MgSO_4_, 4 mM EGTA and 1 mM DTT, pH 7.8). Firefly luciferase activity was measured in a plate luminometer LB960 CentroXS3 (Berthold technologies, Bad Wildbad, Germany) by mixing 20 μl lysates with 72 μl Firefly luciferase assay buffer [25 mM glycyl-glycine (pH 7.8), 15 mM KPO4 (pH 7.8), 15 mM MgSO_4_, 4 mM EGTA, 1 mM DTT and 2 mM ATP (pH 7.6)] and 40 µl of Firefly luciferase substrate (0.2 mM D-luciferin in 25 mM glycyl-glycine) in a white luminometer 96-well plate.

### Cell culture-derived HCV (HCVcc) stock preparation and infection

The reporter viruses were produced by in vitro transcription of the respective plasmid DNA encoding the structural proteins of the respective HCV subtype and the replication complex of the GT2a-derived JFH1 isolate. All inter-genotypic HCV chimeras encode for a luciferase reporter gene, 1b/Con1, 1b/J4, 2a/Jc1, 2b/J8, 3a/S52, 4a/ED43 and 5a/SA13 encode for a Renilla luciferase, whereas the 1a/H77 chimera expresses a secreted Gaussia luciferase. First, 20 μg of plasmid DNA was linearized using MluI restriction enzyme (New England Biolabs, Massachusetts, USA) with the respective buffer and incubated for 1 h at 37 °C. Effective linearization was checked by agarose gel electrophoresis. The DNA was extracted and the concentration determined using Nanodrop equipment. In vitro synthesis of RNA was performed by mixing 2 μg of the purified DNA with 8 μl of T7-RNA Polymerase, 20 μl 5xRRL buffer (400 mM Hepes (pH 7.5), 60 mM MgCl_2_, 10 mM spermidine and 200 mM DTT), 12.5 μl rNTP solution (25 mM of each NTP) and 2.5 μl RNase inhibitor, and incubated for 2 h at 37 °C. Subsequently, 4 μl of T7-polymerase was added and the reaction was incubated for a further 2 h. RNase-free DNAse (Roche, Basel, Switzerland) was added and incubated for 30 min at 37 °C to digest the remaining DNA. Finally, the RNA was purified and the integrity analyzed using agarose gel electrophoresis. RNA concentration was determined and the aliquots were frozen at − 80 °C ready for transfection.

To produce the viral stock, in vitro-transcribed HCV RNA was electroporated into Huh-7.5 cells. To this end, 6 × 10^6^ cells were re-suspended in cytomix solution (2 mM ATP, 5 mM glutathione, 120 mM KCl, 0.15 mM CaCl_2_, 10 mM K_2_HPO_4_/KH_2_PO_4_ (pH 7.6), 25 mM HEPES, 2 mM of EGTA and 5 mM MgCl_2_). The cell suspension was mixed with 5 μg of RNA in an electroporation cuvette with a gap width of 0.4 cm (BioRad). Electroporation was performed in a Gene Pulser (BioRad) at 270 V and 975 μF. Cells were immediately transferred into 16 ml of complete DMEM and seeded on two 10-cm dishes. The supernatant containing HCVcc was harvested at 48 h, 72 h and 96 h post-electroporation and filtered through a 0.45-μm pore-size filter to remove cellular debris. The supernatants were combined and subsequently stored in aliquots at − 80 °C.

For infection assays, Lunet N#3 cells expressing different hCD81 variants were seeded at a density of 0.8 × 10^4^ cells/well of a 96-well plate. The cells were infected 24 h post-seeding with 30 μl of HCVcc-containing supernatants in triplicates. After 4 h of incubation, 170 µl/well of DMEM was added. Plates were then incubated at 37 °C for 72 h. After 72 h of incubation at 37 °C, cells infected with Renilla luciferase expressing HCVcc were lysed using 35 μl of milli-Q water per well and frozen at − 80 °C to ensure complete lysis. Supernatants from infected cells were used for Gaussia luciferase measurement in the case of the 1a/H77/G2a HCVcc chimera. To determine infectivity, luciferase activity was measured using a microplate reader Centro XS (Berthold Technologies). This was done by mixing 20 µl of sample with 60 μl of luciferase substrate solution (Coelenterazine, 0.42 mg/ml in methanol) in 96-well white plates (Berthold).

In cholesterol-depletion experiments, Lunet N#3 cells were pre-incubated with methyl-β-cyclodextrin (MßCD) (Merck) at a concentration of 0.5 mM 30 min before the infection. Medium containing MßCD was removed and the viral inoculum was added. The following infection was performed as mentioned above.

### HCV subgenomic replicon assay

Similar to the HCV reporter viruses, HCV subgenomic replicons were produced by in vitro transcription. To assess the HCV replication efficacy in cell lines expressing different CD81 variants, 5 μg of viral subgenomic RNA (either Luc-NS3-3′/JFH1 or Luc-NS3-3′/JFH1∆GDD) harboring a luciferase reporter gene was transfected into the target cells via electroporation. Luciferase activity as a measure of replication was determined in the cell lysates 4, 24, 48 and 72 h after RNA transfection.

### CD81 structure modelling

A complete structural model of hCD81, including the EC1 loop, created based on the X-ray crystal structure (PDB ID: 5TCX) [27], has been obtained by generating 100 loop-refined models using MODELLER 9.20 [31] and choosing the best model based on the DOPE-HR score implemented in MODELLER and visual inspection. The five side-chain substitutions due to the SNPs were introduced and modeled using MODELLER as well.

### HCV E2 ectodomain structure modelling

The structural figure of the HCV E2 ectodomain was prepared with PyMOL (The PyMOL Molecular Graphics System, Version 1.8.0.3, Schrödinger, LLC) using the E2 ectodomain structure of a genotype 1b09 stain (PDB: 6MEI).

### E2 alignment

The E2 amino acid sequences of the HCV genotypes 1b/Con1, 1b/J4, 2a/JC6, 2b/J8, 4a/ED43 and 5a/SA13 were obtained from UniProt database (https://www.uniprot.org/), and for the sequence of 3a/S52, the NCBI database (https://www.ncbi.nlm.nih.gov) was used. Sequence alignment was performed using the Clustal Omega alignment tool from uniprot [32].

### Statistics

Experiments were performed at least in three biological replicates, each carried out in technical triplicates unless otherwise specified. Results are plotted as mean plus standard deviation (SD) or standard error of mean (SEM) of three biological replicates unless otherwise indicated. Statistical analyses were performed using one-way analysis of variance (ANOVA), followed by Dunnett’s multiple comparison test in GraphPad Prism 8 (GraphPad Software, Inc., San Diego). A *p* value of less than 0.05 was considered statistically significant.

## Results

### Coding non-synonymous SNVs in CD81 regions outside the HCV-binding loop

**Fig. 1:**
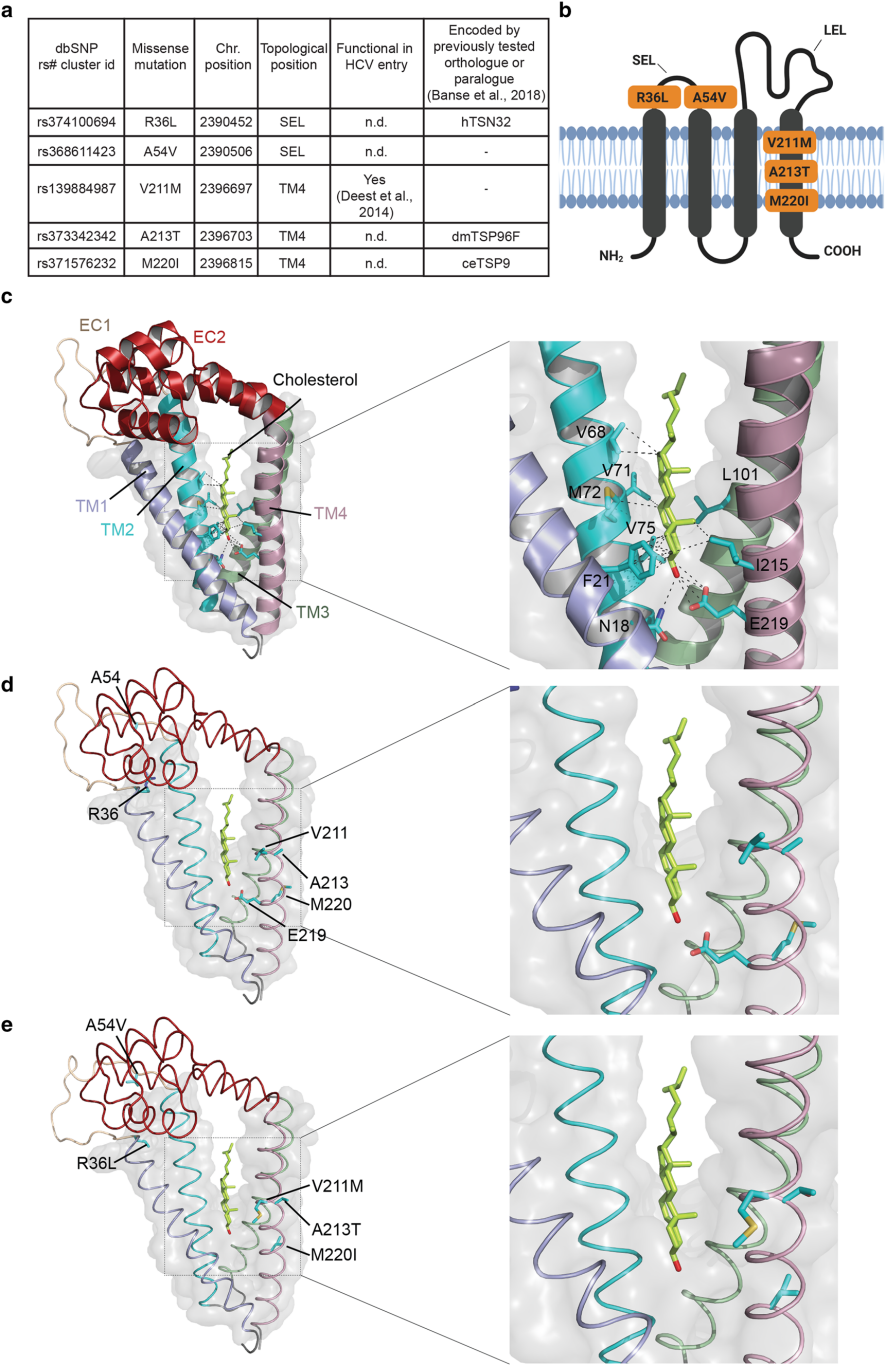
Non-synonymous variants of hCD81 analyzed in this study. **a** SNP database ID number, caused missense mutation and topological position (SEL: small extracellular loop; TM4: transmembrane domain 4) of affected amino acid are listed for the analyzed variants; n.d. not previously determined. **b** Schematic representation of hCD81 with the respective localization of amino acid substitutions due to the five non-synonymous SNVs highlighted (illustration created using biorender.com). **c**, **d** Structure of WT hCD81 according to PDB ID: 5TCX and **e** modelling of the five SNVs constructs. The amino acid exchanges A213T, V211M and M220I localize to the cholesterol-binding pocket in close vicinity to the previously identified cholesterol coordinating residue E219. Right panels show a close-up of the cholesterol-binding pocket

To investigate the effect of different human genetic variants of hCD81 during HCV infection, we focused on five missense mutations that result in the substitution of single amino acids in the non-LEL domains of hCD81. Specifically, these variants which have been reported in the dbSNP database (https://www.ncbi.nlm.nih.gov/snp/) are R36L, A54V, V211M, A213T and M220I (Fig. 1). Variants R36L and A54V are located in the SEL, while variants V211M, A213T and M220I are located in the TM4 domain close to the cholesterol-binding pocket. Three of them are—among other variations—encoded by paralogue or orthologue chimeras previously investigated by us [26], and one of them has been studied by Deest et al. [21]. Furthermore, we also included a sixth construct containing all five SNVs mentioned above. As a negative control, we added the CD81 mutant F186A, which binds poorly to E2 due to an amino acid exchange in the LEL [33].

### Expression of hCD81 variants in human hepatoma cells

**Fig. 2:**
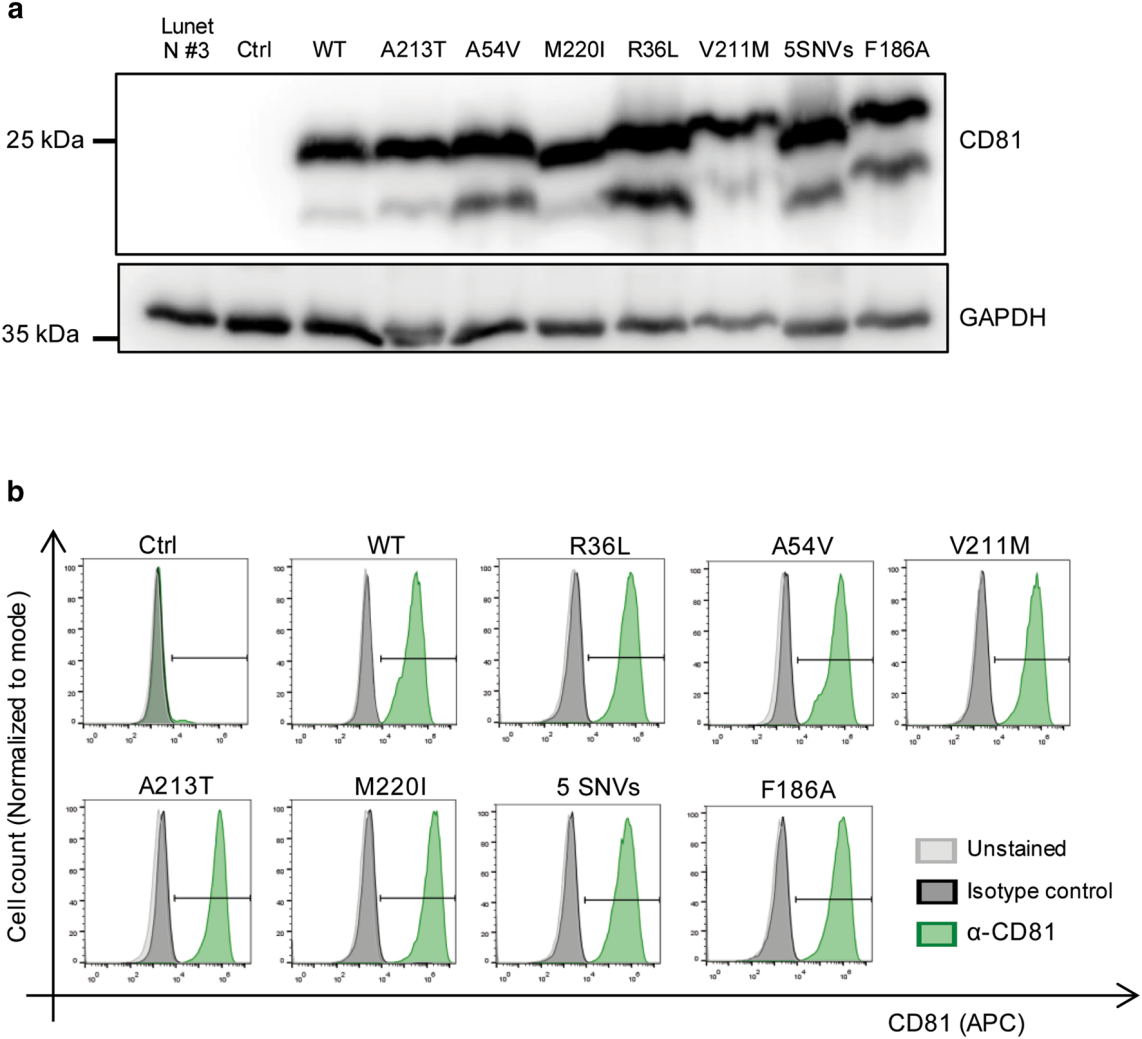
hCD81 variants and WT hCD81 translocate to the cell surface of human hepatoma cells. **a** Immunoblot of cell lysates from Lunet N#3 cells transduced with lentiviral pseudoparticles harboring the different hCD81 variants, WT hCD81 or control vector and probed with anti-CD81 antibody. GAPDH served as loading control and Lunet N#3 cells as negative control. **b** Cell surface expression of hCD81 assessed by flow cytometry after staining with anti-hCD81-APC antibody. An APC-conjugated isotype control antibody or buffer only served as negative controls. Representative histograms comparing anti-hCD81-APC-stained, isotype control-stained and unstained cells from all cell lines included in this study. The histograms show one out of two independent experiments with 2.0 × 10^4^ cells per measurement. The gate for hCD81-positive cells is shown as horizontal line. Representative histograms of two independent biological replicates are shown

**Fig. 3:**
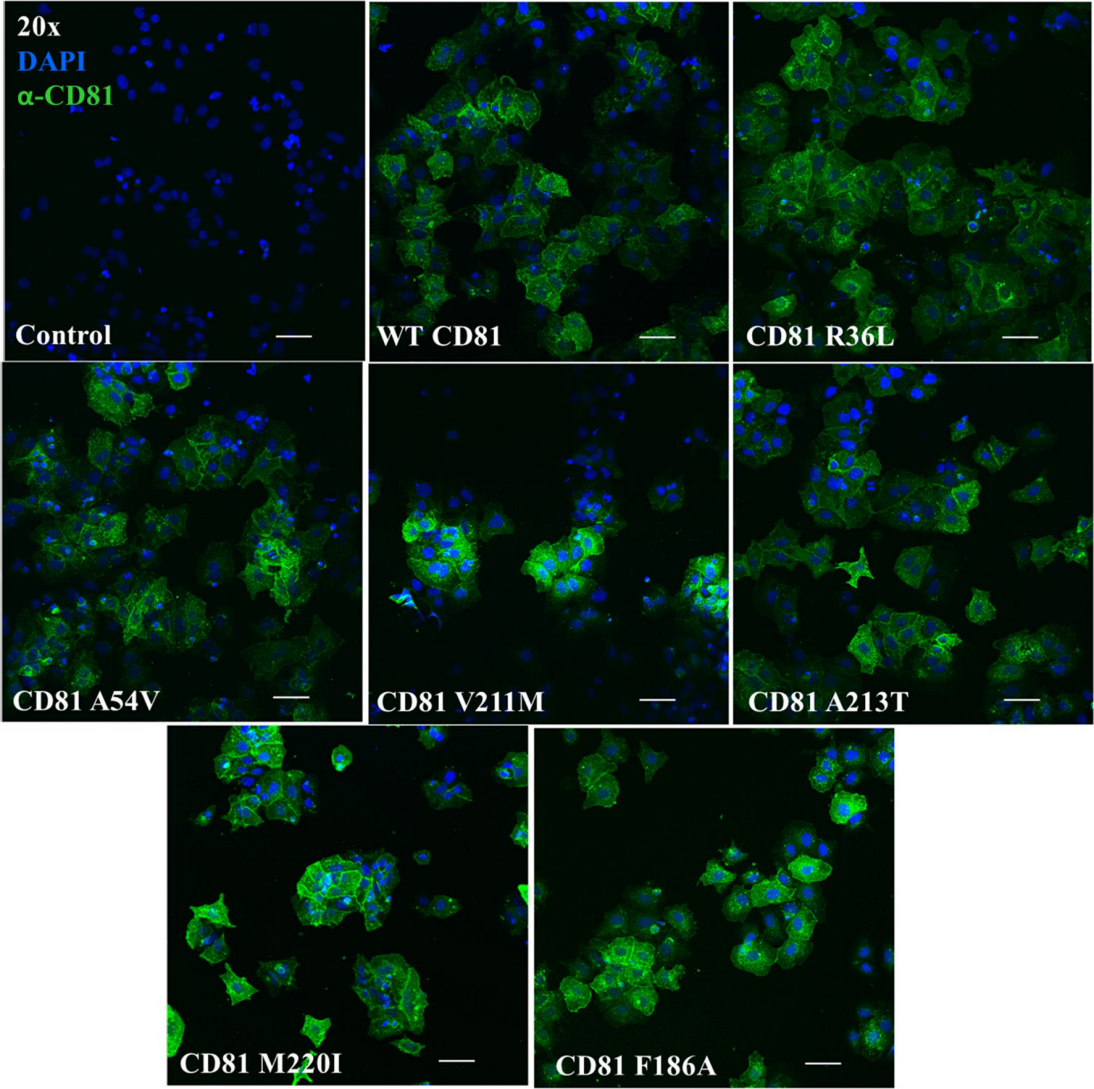
hCD81 variants and WT hCD81 localize to similar subcellular compartments in human hepatoma cells. hCD81 variant-expressing Lunet N#3 cells were fixed, permeabilized and stained with anti-hCD81 antibody (Clone JS-81, green). Hepatoma cells expressing WT hCD81 and lacking hCD81 served as positive and negative controls, respectively. Nuclei were stained with DAPI (blue). Representative confocal microscopy images of a single plane; magnification: × 20, scale bars 10 μm

To test the effect of the hCD81 SNVs on HCV susceptibility, we generated cell lines stably expressing the different hCD81 variants. To this end, we transduced lentiviral pseudoparticles harboring the different hCD81 variants into Lunet N#3 cells, known to have undetectable levels of endogenous hCD81 [28]. We named the generated cell lines by the missense mutation that they carried: R36L, A54V, V211M, A213T, M220I and 5 SNVs. Transduction with empty vector (Ctrl) and WT hCD81 constructs served as negative and positive controls, respectively. First, we assessed the CD81 expression in whole cell lysates by immunoblotting using anti-CD81 antibody. As expected, Lunet N#3 cells and cells transduced with empty vector did not show any band corresponding to CD81. Conversely, cells transduced with WT CD81 and all hCD81 variants showed a band at 26 kDa, the molecular weight (MW) of CD81. Additional bands at lower MW were detected in WT CD81 and all variants, which correspond to degradation products or splice variants of CD81 since they were not present in Lunet N#3 cells and cells transduced with empty vector (Fig. 2a). Next, we determined the cell-surface expression of hCD81 in single-cell suspensions by flow cytometry using an anti-hCD81-APC antibody. As expected, Lunet N#3 cells transduced with empty vector do not express detectable levels of hCD81 on the cell surface. WT hCD81 and all the hCD81 variants were expressed on the cell surface at a comparable level as measured by mean fluorescence intensity (MFI) (Fig. 2b, Fig. S1a), thereby confirming trafficking of hCD81 to the plasma membrane. Additionally, each of the cell lines showed a transduction efficiency higher than 98% (Fig. S1b). Finally, we studied the subcellular distribution of the hCD81 variants by immunofluorescence staining followed by confocal microscopy. To that end, we fixed cells, permeabilized and stained them with anti-hCD81 antibody. We stained the nuclei with DAPI. Using confocal microscopy, we observed that all hCD81 variants and WT hCD81 displayed similar staining patterns with intracellular localization as well as plasma membrane localization (Fig. 3).

### hCD81 variant-expressing cells show comparable susceptibility to HCV pseudoparticles

**Fig. 4:**
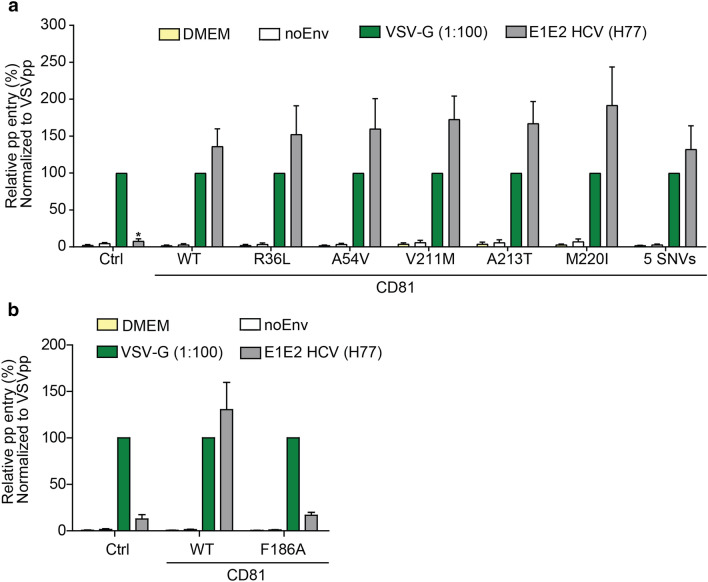
hCD81 variants function as receptors for lentiviral HCV pseudoparticles similar to WT hCD81. Lunet N#3 cells expressing different hCD81 variants (**a**) or the non-binding mutant F186A (**b**) were challenged with lentiviral particles displaying the E1E2 glycoproteins from HCV strain H77 (genotype 1a) on their surface. Cells lacking hCD81 served as an internal control. Pseudoparticles displaying VSV-G envelope glycoprotein and pseudoparticles without envelope glycoproteins (noEnv) served as positive and negative controls, respectively. All pseudoparticles carried a firefly luciferase reporter gene. Firefly luciferase activity was measured at 72 h post-transduction. HCVpp infectivity was plotted relative to VSV-G-mediated entry (%). Mean + SEM of four independent biological replicates each performed in technical triplicate shown

To determine the impact of the SNVs on the CD81 receptor function, we infected the cells expressing hCD81 variants with lentiviral pseudoparticles presenting HCV envelope glycoproteins E1 and E2 from strain H77 (genotype 1a). As a positive control, we used pseudoparticles bearing the glycoprotein of vesicular stomatitis virus (VSV-G). Pseudoparticles with no envelope glycoproteins (noEnv) served as a negative control. All pseudoparticles encoded a Firefly reporter gene to allow the quantification of infected cells by measuring Firefly luciferase (FLuc) activity in relative light units (RLU) per well. As expected, VSV-G pseudoparticles efficiently infected every cell line. To compare the susceptibility of the different cell types to HCV pseudoparticles (HCVpp), we normalized the HCVpp infection to that of VSVpp (Fig. 4). Cells without CD81 (Ctrl) showed an HCVpp infectivity of 8% relative to VSV and represented the background infectivity level. Similarly, the pseudoparticles with no envelope proteins (negative control) showed an infectivity of less than 7% relative to VSV in all the cases confirming this background level. The WT CD81 and all hCD81 variant-expressing cells similarly supported HCVpp entry with no significant differences. As expected, the CD81 F186A mutant, which fails to bind HCV E2, did not render cells HCVpp susceptible (Fig. 4b). These results suggest that the presence of the SNVs in hCD81 does not affect its function as HCV receptor for lentiviral HCVpp, which partially mimics authentic viral entry.

### hCD81 variant-expressing cells are differentially permissive to cell culture-derived HCV (HCVcc)

**Fig. 5:**
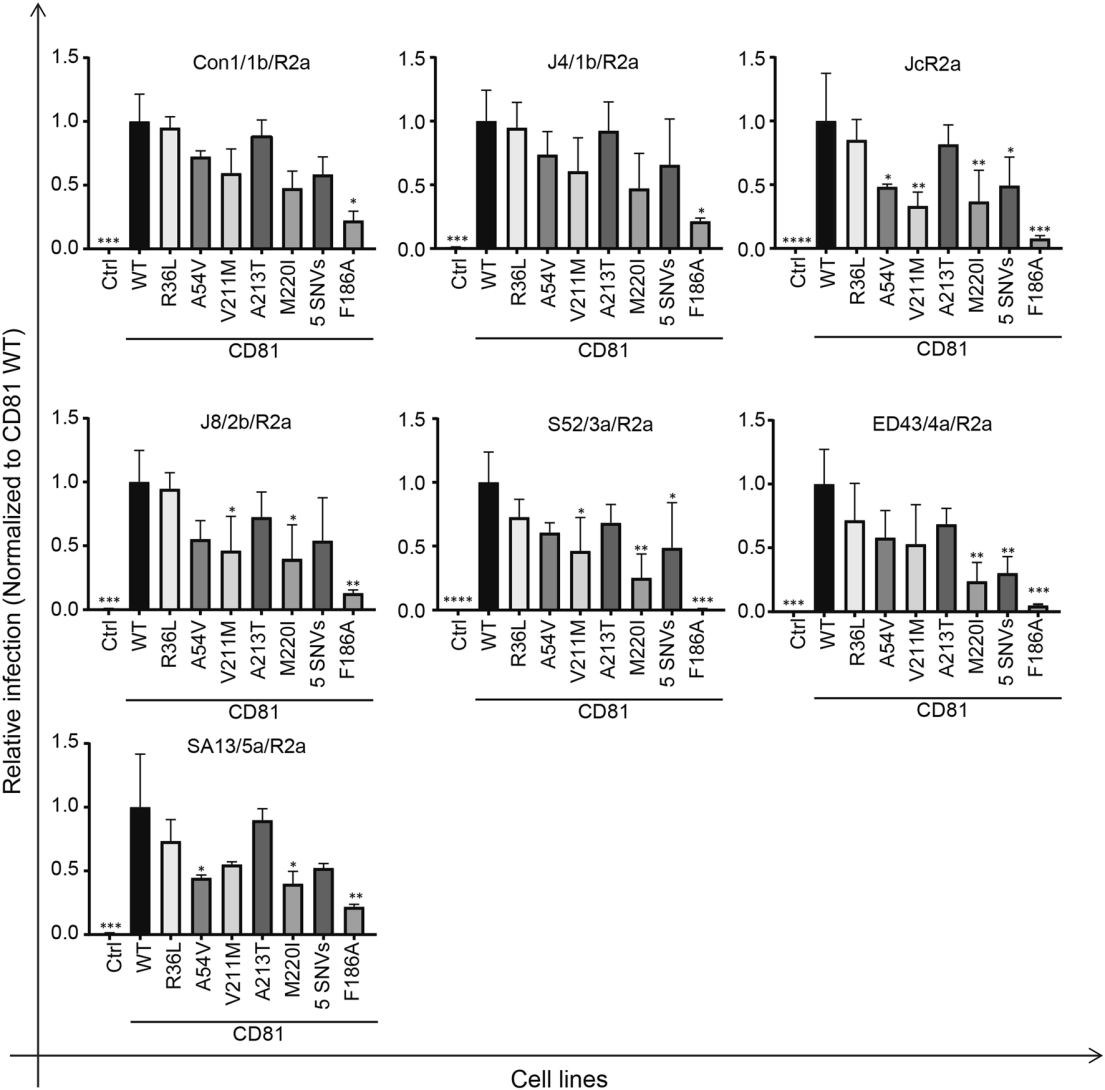
A subset of hCD81 variants reduces HCV susceptibility of human hepatoma cells. hCD81-variant expressing cells were challenged for 4 h with cell culture-derived HCV chimeras encoding the glycoproteins of the seven indicated genotypes as well as Renilla luciferase. Luciferase activity in cell lysates was measured 72 h post-infection and the results were plotted relative to infection of cells expressing WT CD81. Mean + SD of three independent biological replicates each performed in technical triplicates shown (GT5a and F186A variant experiments performed twice in technical triplicates). *p < 0.05, **p < 0.01, ***p < 0.001, ****p < 0.0001

Since HCVpp assays only mimic certain steps of HCV entry, we next performed HCVcc assays to test the effect of the different hCD81 variants on an authentic HCV infection. To that end, we infected hCD81 variant-expressing cells with seven inter-genotypic HCV chimeras: Con1/1b/R2a, J4/1b/R2a, JcR2a, J8/2b/R2a, S52/3a/R2a, ED43/4a/R2a and SA13/5a/R2a. These chimeric viruses encode the structural proteins of genotypes 1 through 5 allowing the study of genotype-specific infection events [34, 35]. The complete panel of viruses encoded a Renilla luciferase reporter gene. We thus determined HCV infectivity at 72 h post-infection by measuring luciferase activity in cell lysates. Measurement windows were comparable for all seven HCVcc strains (Fig. S2a). We plotted HCVcc infection of the different cell lines normalized to the infection of the cell line expressing WT hCD81. As expected, cells without hCD81 (Ctrl) displayed background luciferase activity after HCVcc infection with every tested genotype including a H77 (GT 1a) strain, which expressed a Gaussia luciferase reporter gene (Fig. S2b). Similarly, and in accordance to the HCVpp assay, cells expressing the E2 binding-deficient F186A hCD81 mutant showed reduced infection with all seven Renilla genotypes tested. For the genotype 3a-S52 and 4a-ED43 chimeras, the infectivity was as low as in the control cells. Of the novel hCD81 variants tested, the variants R36L and A213T conferred similar susceptibility to Lunet N#3 cells as WT hCD81 to every genotype tested. In contrast, variants A54V, V211M and M220I conferred reduced susceptibility to HCV. The A54V variant showed a twofold reduced ability to support HCVcc infection compared to WT hCD81 when the cells were challenged with the genotype 2a and 5a chimeric viruses. Cells expressing the V211M variant supported HCVcc infection three-, two- and twofold less efficiently than WT hCD81 for genotype 2a-Jc1, 2b-J8 and 3a-S52 chimeric viruses, respectively. Variant M220I showed the strongest impairment in HCVcc infection. Infection with five inter-genotypic chimeras (2a-Jc1, 2b-J8, 3a-S52, 4a-ED43 and 5a-SA13) was significantly lower than the WT hCD81 showing 3-, 2.5-, 4-, 4- and 2.5-fold reduction, respectively. Finally, 5 SNV-expressing cells showed two-, two- and threefold reduction of susceptibility to HCVcc infection with genotypes 2a-Jc1, 3a-S52 and 4a-ED43, respectively, as compared to cells expressing WT hCD81 (Fig. 5).

The hCD81 variants V211M and M220I supported HCV infection less efficiently than WT hCD81. Due to the close proximity of the altered amino acid residues to the cholesterol binding pocket of hCD81, we investigated if cholesterol depletion would affect the performance of the hCD81 variants as HCV entry factor. To this end, we pre-incubated Lunet N#3 cells expressing hCD81 WT, hCD81, V211M and M220I with 0.5 mM methyl-β-cyclodextrin (MßCD) before HCVcc infection. Independent of the expressed hCD81 variant, all cells showed the same percentage of reduction in the susceptibility to GT 2a, 2b and 3a upon treatment with MßCD (Fig. S3).

To determine if the hCD81 variants affect a post-entry step of the HCVcc reporter assay, we performed replication assays using subgenomic reporter replicons. After transfecting the in vitro-transcribed HCV subgenomes into the *hCD81* SNV- and WT-expressing cells, we did not observe any difference in replication efficiency (Fig S4a). As expected, transfection of a replication-deficient subgenome did not result in replication measured by luciferase activity (Fig S4b). Thus, the hCD81 variants do not alter the cell’s ability to replicate HCV genomes.

We observe that some of the genotypes appear more sensible to the modifications in the backbone amino acid sequence of hCD81 than others: four of the six variants tested affect the infectivity of genotype 2a. The infectivity of genotype 3a-S54 is reduced in the presence of three hCD81 variants, whilst genotypes 2b-J8, 4a-ED43 and 5a-SA13 are affected by two hCD81 variants. Finally, the infectivity of genotype 1b is not significantly affected by the presence of any hCD81 variant included in this study. We, therefore, compared the HCV E2 amino acid residues, described to contribute to hCD81 binding [36–39] for the seven HCV genotypes (Fig S5a). While all the genotypes consistently display large hydrophobic aromatic amino acids in the four critical hCD81-binding regions, the hCD81 SNV-sensitive genotypes harbour an amino acid variation at position 444 in E2 region 4. Specifically, the tested genotype 1b strains harbour a non-polar amino acid (Ala or Val) at position 444, and the hCD81 SNV-sensitive genotypes (2a, 2b, 3a, 4a, 5a) harbour moderately polar amino acids (Tyr, Thr) (Fig. S5b). Taken together, these results show that three of the five tested hCD81 variants function less efficiently as HCV host factors for specific viral genotypes.

## Discussion

Hepatitis C virus entry into the hepatocytes comprises a complex multistep process which involves the interaction with at least four host factors: CD81, SBR1, CLDN1 and OCDN [4–7]. CD81 is implicated in HCV binding [5, 36], lateral translocation [40], endocytosis [41] and HCV priming for low pH-dependent fusion with endosomal membranes [42] during the entry process. Thus, CD81 is an interesting candidate to study species barriers or the impact of naturally occurring hCD81 SNVs towards HCV infectivity.

While the LEL of CD81 mediates HCV E2 binding [5], the function of the CD81 domains outside the LEL backbone in the HCV entry is less well characterized. Banse et al., demonstrated using chimeric CD81 that the backbone domains play a role during the post-binding steps, being critical for productive HCV entry [26]. The specific residues in the backbone that are important for HCV uptake remain poorly understood although a cholesterol-coordinating residue (E219) seems important [26, 43]. In this study, we aimed to investigate the effect of five non-synonymous SNVs, specifically R36L, A54V, V211M, A213T and M220I, on HCV infectivity. In addition, we included a CD81 construct, which harbours all five SNVs. The selected SNVs result in amino acid changes in the SEL (R36L and A54V) and in the TM4 domain (V211M, A213T and M220I) of hCD81. We used Lunet N#3 cells, which have undetectable levels of endogenous CD81 [28], to generate new cell lines expressing six different hCD81 variants. Afterwards, we examined the expression of the variants and their functionality as HCV receptor. By LEL-directed antibody staining, flow cytometry and confocal microscopy, we observed that all hCD81 variants are expressed at the cell surface to comparable levels. Moreover, as we used a conformation-sensitive antibody, our results suggest that the LEL is properly folded. Further binding assays with a soluble HCV E2 glycoprotein on Chinese Hamster Ovary 745 cells (CHO745) [44] expressing the hCD81 variants could be performed to confirm proper folding of the virus-binding ectodomain. Regarding the functionality of the hCD81 variants as HCV entry factors, we demonstrated that all variants similarly support entry of HCVpp with glycoproteins of genotype 1a (H77). Nevertheless, three of the five tested variants, namely A54V, V211M and M220I, are less susceptible to infection with HCVcc genotypes 2, 3, 4 and 5 in comparison to WT CD81-expressing cells.

### Effect of different hCD81 variants on HCVpp entry

The lentiviral pseudoparticle system (HCVpp) mimics certain aspects of infectious HCV entry including entry-factor interactions, thereby being an important tool to study the cell-surface binding step of the HCV life cycle. However, this technique has some limitations with regard to the particle architecture, binding to serum lipoproteins and capsid origin. The lentiviral particles are bigger in size (100 nm vs 60 nm) and display a lower density of E1/E2 dimers in comparison to serum-derived HCV particles [45]. These differences in the architecture of lentiviral particles compared to HCV particles may lead to differences in endocytosis and fusion processes. For instance, we identified three CD81-associated proteins, CAPN5, CBLB and SRFBP1, which are required for HCV entry. CAPN5 and CBLB are involved in endocytosis whereas SRFBP1 is required in an endocytosis-independent post-binding step of HCV entry. These proteins are exclusively host factors for HCVcc, but not HCVpp [24, 25]. Common features of HCVcc and HCVpp uptake are the use of the clathrin-mediated endocytosis machinery [8, 46]. This also holds true for HCVcc entry into 3D organoid cultures [47]. Moreover, unlike serum-derived and cell culture-derived HCV particles, lentiviral particles cannot bind to serum lipoproteins. This limits the mimicry of HCVpp cell-surface attachment via LDL-R and SCARB1, and may affect lipid-transfer functions necessary for productive HCV entry [48, 49]. Finally, this system does not allow the study of HCV uncoating since HCVpp comprises a lentiviral capsid [45].

In this study, we show that HCVpp entry efficiency remains unaltered by hCD81 variants when testing lentiviral particles decorated with glycoproteins from genotype 1a (H77). Further studies, including pseudoparticles expressing glycoproteins from other HCV genotypes, will help to determine if hCD81 variants affect HCV binding in a genotype-dependent manner. Grove et al. showed an excellent agreement between their J6 HCVpp and J6/JFH HCVcc assays when comparing single- and double-amino acid exchange mutants, which affect the conformation of CD81 [43]. In our HCVpp assay, the entry step was not affected by the different hCD81 variants, which is in contrast to the results from our HCVcc assay. It remains to be shown if the discrepancy observed between HCVpp and HCVcc is attributed to the different genotypes used or due to the differences in the entry process of lentiviral pseudotypes, and cell culture-produced HCV. Interestingly, the hCD81 variant-sensitive E2 proteins from genotypes 2, 3, 4, and 5 harbour an additional polar amino acid in one of the four hCD81-binding regions. This may suggest that these E2 proteins have a reduced hydrophobic-binding patch and less efficiently engage hCD81. If hCD81 backbone variants alter the hCD81 ectodomain conformation, differential binding efficiencies of the specific HCV genotypes other than genotype 1 could occur. In summary, we conclude that the tested hCD81 SNVs do not affect HCV GT1a E2 binding to hCD81, which is reliably mimicked by the HCVpp system.

### Effect of different hCD81 variants on HCVcc infection

To analyze the effect of the hCD81 SNVs on HCV entry steps beyond E2 binding, we performed HCVcc infection assays. HCV is classified into seven distinct genotypes which are differentially distributed globally [50]. HCV genotypes differ considerably in their glycoprotein sequences with a divergence between 30 and 35% at the nucleotide level [51]. This divergence leads to qualitative and quantitative differences in virus–receptor interactions and viral neutralization capacities by antibodies [52, 53]. In our study, we included five different HCV genotypes (1–5) and subtypes to elucidate if *hCD81* variants affect the authentic HCV infection in a genotype-specific manner. We used inter-genotypic chimeric viruses [34, 54]. The chimeric viruses encode structural proteins (Core, E1, E2), NS2 and p7 specific for the respective genotype, and the replication complex from a genotype 2a JFH-1 isolate (NS3, NS4A, NS4B, NS5A and NS5B) [34, 35]. Thus, the observed genotype-specific differences are attributed to the structural proteins and not the viral replication machinery. We show that hCD81 variants A54V, V211M and M220I rendered the cells between two and fourfold less susceptible to HCV infection for specific viral genotypes, such as 2, 3, 4 and 5, compared to WT hCD81-expressing cells. In contrast, no significant protection against HCV infection was conferred by R36L and A213T amino acid substitutions. Zimmerman et al. recently demonstrated that conformational changes, i.e. opening of EC2, upon binding of cholesterol to the transmembrane domain are crucial to hCD81 function [27]. Interestingly, variants V211M and M220I are located in TM4, next to the cholesterol-binding pocket, whereas A213 in TM4 is facing the lipid bilayer and not involved in cholesterol interactions (Fig. 1). By depleting cholesterol using MβCD, we demonstrated that WT hCD81 and variants V211M and M220I require cholesterol to the same extent to function as HCV entry factors (Fig. S3). Thus, the amino acid changes at position V211 and M220 seem not to affect the cholesterol coordination by hCD81, which is required for HCV infection [26]. We speculate that A54V may alter cholesterol-dependent dynamics of extracellular EC2, but definite conclusions on structure–function relationships of these mutants await further research. Interestingly, variant V211M was studied before and shown to support HCVcc entry similar to WT hCD81 [21]. This discrepancy could be explained by the differences in the methodological procedure. Deest et al. infected Lunet N4 cells with F-Luc Jc1 virus for 5 h, and luciferase activity was measured 48 h later. Despite studying the same genotype (2a), we infected Lunet N#3 cells with R-Luc JcR2a virus for 4 h and the luciferase activity was determined 72 h later. Possibly, our readout system was more sensitive to detect the threefold differences in the entry efficiency. Notably, we observed reduced HCVcc infectivity in the variant V211M compared to WT hCD81 for three of the seven genotypes tested.

CD81 is involved not only in viral infection, but also in cell biological processes. The physiological function of CD81 in hepatoma cells is poorly defined. In the immune cells, CD81 is involved in cell adhesion, motility, activation, proliferation, differentiation and signal transduction [14]. To confirm that the reduction in HCV susceptibility observed in HCVcc experiments was due to the receptor function of hCD81 variants and not general impairment in morphology or cell growth, it would be important to perform infection assays with hCD81-independent enveloped viruses, such as VSV and human coronavirus. However, as we neither observed differences in susceptibility to HCVpp nor in the susceptibility to the HCVcc genotype 1b, our data strongly suggest that the observed differences in HCV susceptibility are attributed to a differential entry-factor function of the respective hCD81 SNVs and not unspecific effects on cellular functions.

The HCVcc infection assay used in this study reflects several virus life cycle steps including entry, translation and genome replication. In addition to its role in HCV entry, CD81 was shown to be necessary for effective viral replication [55]. To uncouple the entry from the translation and replication step, we performed subgenomic replicon assays. Translation and replication of the tested luciferase reporter replicons were comparable in all tested cells lines. This strongly suggests that the differences observed in the HCVcc assay is attributed to differential functions of the *hCD81* variants in virus entry.

Variants A54V, V211M and M220I did not impair HCVpp entry but rendered Lunet N#3 cells less susceptible to infection with specific HCV genotypes in HCVcc assays compared with WT hCD81 expressing cells. We, therefore, hypothesize that a post-binding step could be affected by hCD81 variants. CD81 interacts with several host proteins after HCV-LEL binding [23, 24, 56, 57], and these interactions are important for the productive HCV uptake. Co-immunoprecipitation assays, comparing protein interactions of WT and variant hCD81, will help to elucidate if the identified hCD81 variants alter important protein–protein interactions, thereby expanding the knowledge about the hCD81 backbone function.

Having demonstrated that some SNVs affect HCV infection in vitro and considering that these genetic variants are present in the population, it would be interesting to perform cohort studies. Such studies will allow us to determine if the results obtained in vitro are also observed in vivo in the population. In a previous study, variants of SR-BI were shown to affect HCV viral load in patients [13]. Investigation of SNPs may help predict individual susceptibility, disease progression and/or response to antiviral treatment. The SNVs investigated in this study have very low frequency in the human population. The Genome Aggregation Database reported frequencies of 0.0004% for R36L, 0.003% for A54V, 0.01% for V211M, 0.002% for A213T and 0.0004% for M220I in the human population. Therefore, we decided to first perform the in vitro studies to evaluate if searching for those rare individuals with a specific SNV in HCV cohorts will provide important information. After identifying three hCD81 SNVs with an impact on GT2 infection, we consider to genotype the three SNVs in GT2 patient cohorts and in highly exposed individuals, which control the HCV infection. Due to the low frequency of the SNVs, such investigation will likely not reach statistical significance. Nonetheless, it may provide important data on whether hCD81 SNVs can contribute to a better control of HCV infection. Importantly, CD81 is also a host factor for the malarial parasite *Plasmodium* [16] and for influenza A virus [17], two human pathogens affecting hundreds of millions of individuals globally [58, 59]. Whether hCD81 SNVs affect the individual susceptibility to *Plasmodium* or influenza virus is a possibility that we would like to test.

In conclusion, our work provides evidence that the non-synonymous SNVs A54V, V211M and M220I in the backbone of hCD81 render hepatoma cells less susceptible to HCV infection. Our findings enrich the understanding of the inter-individual differences that may govern HCV susceptibility and disease progression. This study further confirms that hCD81 non-LEL domains are involved in the HCV life cycle and identifies specific residues that could be important in HCV post-binding events.

## Electronic supplementary material

Below is the link to the electronic supplementary material.

Supplemental Fig. 1 hCD81 variants and WT hCD81 translocate to the cell surface of human hepatoma cells. Cell surface expression of hCD81 assessed by flow cytometry after staining with anti-hCD81-APC antibody. An APC-conjugated isotype control antibody or buffer only served as negative controls. (a) Mean fluorescence intensity (MFI) of the whole cell population and (b) percentage of positive cells shown as quantification of cell surface hCD81 expression. Mean values + SD of two independent biological replicates are shown (TIF 11618 kb)

Supplemental Fig. 2 Comparison of infectivity of HCV intergenotypic chimeras used in this study in hCD81 WT expressing cells. (a) Shown is the measurement of Renilla luciferase in relative light units (RLU) in cell lysates of infected hCD81 expressing cells and empty vector expressing cells from Fig. 5. (b) hCD81 expressing cells and and empty vector control cells were infected with the chimeric H77/1a/G2a virus encoding a Gaussia luciferase. Secreted Gaussia luciferase was measured 72 hours post infection in supernatants of infected cells. Plotted are the raw data in RLUs. Means +SD of three independent biological replicates in technical triplicates are shown (TIF 196 kb)

Supplemental Fig. 3 Effect of cholesterol depletion on HCVcc infection of hCD81 WT and variant expressing cells. WT hCD81 and variant V211M and M220I expressing cells were pre-treated with 0.5 mM MßCD 30 min before infection. MßCD was removed and HCVcc of the respective chimeras added for 4 hours. Luciferase activity in cell lysates was measured 72 hours post infection and the results were plotted relative to infection of untreated cells. Mean + SD of three independent biological replicates each performed in technical triplicates (TIF 194 kb)

Supplemental Fig. 4 Effect of hCD81 variants on HCVcc replication. hCD81 WT and variant expressing cells were transfected with a replication competent (a) or replication deficient (dGDD) (b) in-vitro transcribed HCV reporter subgenome. Luciferase activity in cell lysates was measured after 4, 24, 48 and 72 hours post transfection and the results were plotted relative to luciferase activity after 4 hours. Graphs show mean + SD of three independent biological replicates each performed in technical triplicates (TIF 393 kb)

Supplemental Fig. 5 (a) Sequence alignment of HCV E2 from the tested genotypes. Regions of neutralizing antibody binding with implications in CD81 interaction are highlighted. Amino acids which differ between hCD81 SNV sensitive and resistant HCV genotypes are marked in red. Included are all tested genotypes as well as the sequence GT1b_09 used for the structural model in (b). (b) Structure of E2 ectodomain of GT1b_09. Regions 1-4 are colored according to (a). Side chains of residues within regions 1-4 which differ between compared HCV genotypes and strains are shown in stick representations with oxygen and nitrogen atoms colored in red and blue, respectively. All four regions include large and strictly conserved hydrophobic, aromatic amino acids (W420, Y443, W529, W616), which are shown in line representations. (TIF 2203 kb)
