# Supplementary figures and images for: Single-nucleotide variants in human CD81 influence hepatitis C virus infection of hepatoma cells
Source: Med Microbiol Immunol. 2020 Apr 22;209(4):499–514. doi: 10.1007/s00430-020-00675-1 (PMC7176029; doi:10.1007/s00430-020-00675-1)

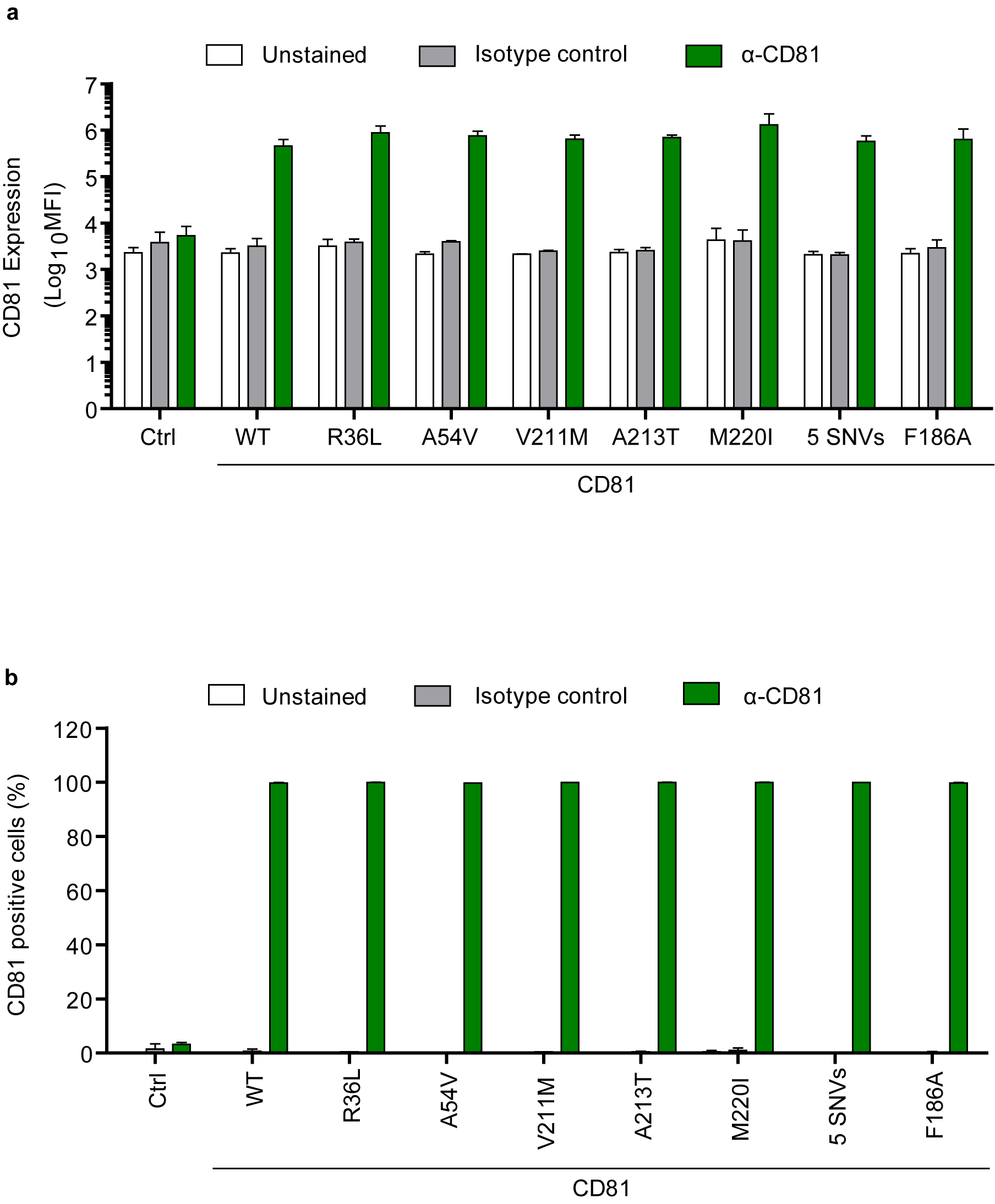

Supplement: Supplementary file 1 — Supplemental Fig. 1 hCD81 variants and WT hCD81 translocate to the cell surface of human hepatoma cells. Cell surface expression of hCD81 assessed by flow cytometry after staining with anti-hCD81-APC antibody. An APC-conjugated isotype control antibody or buffer only served as negative controls. (a) Mean fluorescence intensity (MFI) of the whole cell population and (b) percentage of positive cells shown as quantification of cell surface hCD81 expression. Mean values + SD of two independent biological replicates are shown (TIF 11618 kb) [file 430_2020_675_MOESM1_ESM.tif]

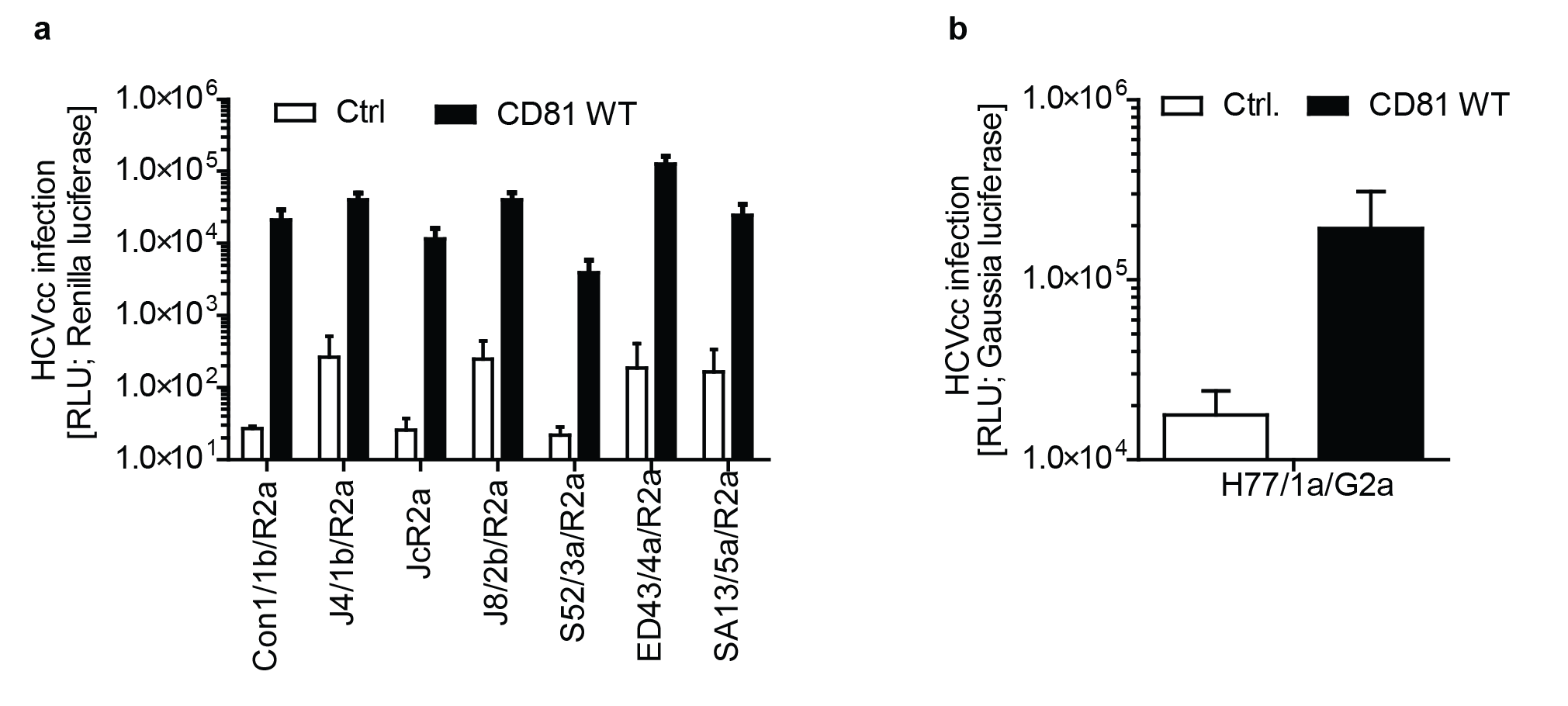

Supplement: Supplementary file 2 — Supplemental Fig. 2 Comparison of infectivity of HCV intergenotypic chimeras used in this study in hCD81 WT expressing cells. (a) Shown is the measurement of Renilla luciferase in relative light units (RLU) in cell lysates of infected hCD81 expressing cells and empty vector expressing cells from Fig. 5. (b) hCD81 expressing cells and and empty vector control cells were infected with the chimeric H77/1a/G2a virus encoding a Gaussia luciferase. Secreted Gaussia luciferase was measured 72 hours post infection in supernatants of infected cells. Plotted are the raw data in RLUs. Means +SD of three independent biological replicates in technical triplicates are shown (TIF 196 kb) [file 430_2020_675_MOESM2_ESM.tif]

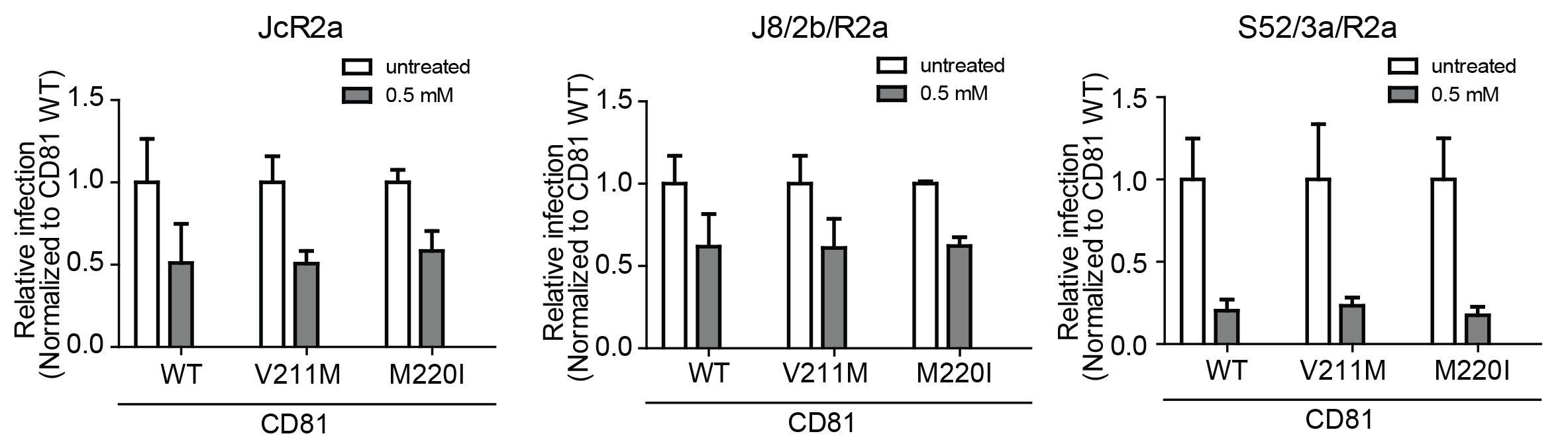

Supplement: Supplementary file 3 — Supplemental Fig. 3 Effect of cholesterol depletion on HCVcc infection of hCD81 WT and variant expressing cells. WT hCD81 and variant V211M and M220I expressing cells were pre-treated with 0.5 mM MßCD 30 min before infection. MßCD was removed and HCVcc of the respective chimeras added for 4 hours. Luciferase activity in cell lysates was measured 72 hours post infection and the results were plotted relative to infection of untreated cells. Mean + SD of three independent biological replicates each performed in technical triplicates (TIF 194 kb) [file 430_2020_675_MOESM3_ESM.tif]

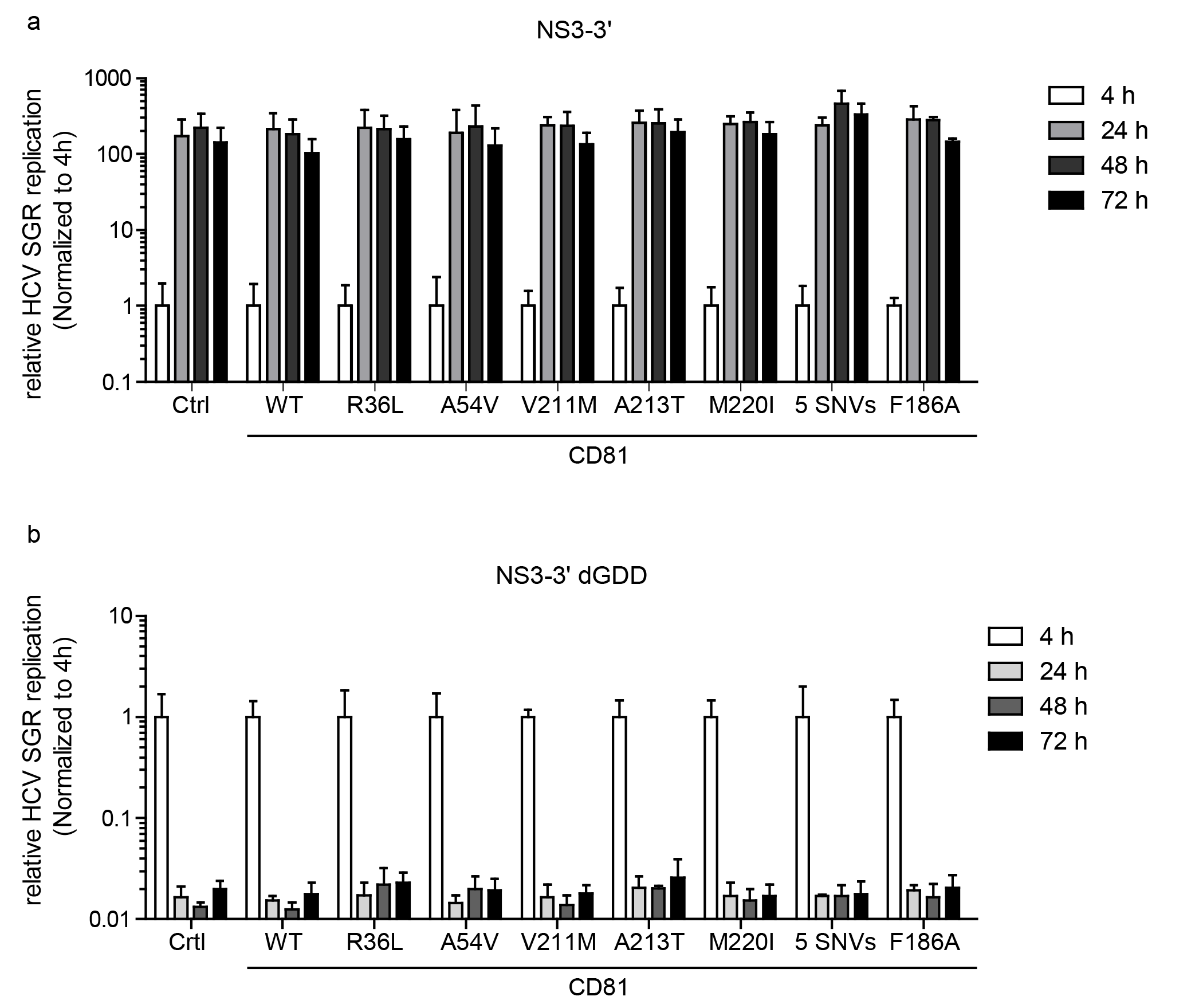

Supplement: Supplementary file 4 — Supplemental Fig. 4 Effect of hCD81 variants on HCVcc replication. hCD81 WT and variant expressing cells were transfected with a replication competent (a) or replication deficient (dGDD) (b) in-vitro transcribed HCV reporter subgenome. Luciferase activity in cell lysates was measured after 4, 24, 48 and 72 hours post transfection and the results were plotted relative to luciferase activity after 4 hours. Graphs show mean + SD of three independent biological replicates each performed in technical triplicates (TIF 393 kb) [file 430_2020_675_MOESM4_ESM.tif]

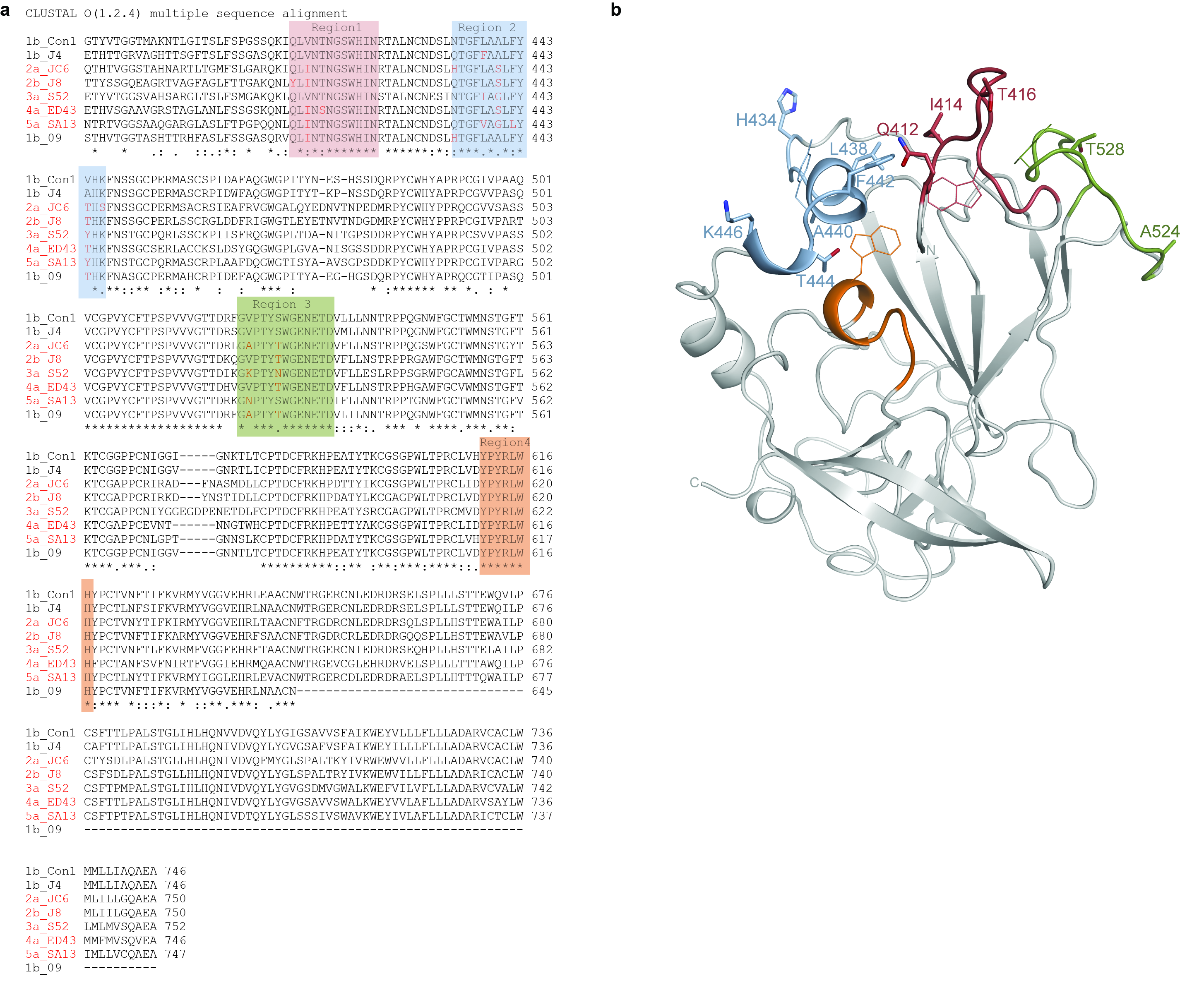

Supplement: Supplementary file 5 — Supplemental Fig. 5 (a) Sequence alignment of HCV E2 from the tested genotypes. Regions of neutralizing antibody binding with implications in CD81 interaction are highlighted. Amino acids which differ between hCD81 SNV sensitive and resistant HCV genotypes are marked in red. Included are all tested genotypes as well as the sequence GT1b_09 used for the structural model in (b). (b) Structure of E2 ectodomain of GT1b_09. Regions 1-4 are colored according to (a). Side chains of residues within regions 1-4 which differ between compared HCV genotypes and strains are shown in stick representations with oxygen and nitrogen atoms colored in red and blue, respectively. All four regions include large and strictly conserved hydrophobic, aromatic amino acids (W420, Y443, W529, W616), which are shown in line representations. (TIF 2203 kb) [file 430_2020_675_MOESM5_ESM.tif]
